# Midwives’ competence and confidence in Kenya: a sequential explanatory study design

**DOI:** 10.1186/s12913-025-13576-x

**Published:** 2026-01-13

**Authors:** Tallam Edna, Kaura Doreen, Mash Bob

**Affiliations:** 1 Department of Nursing and Midwifery, Faculty of Medicine and Health Sciences, Stellenbosch University, Cape Town, South Africa; 2 Amref International University, Northlands, Nairobi, 27691 – 00506 Kenya; 3 School of Nursing, Faculty of Community Health Sciences, University of the Western Cape, Cape Town, South Africa

**Keywords:** Explanatory sequential design, Competence, Confidence, Knowledge, Skills and behaviour, Education, Kenya

## Abstract

**Background:**

The competence and confidence of midwives are pivotal in delivering essential care during pre-pregnancy, pregnancy, childbirth, and postpartum for women and their newborns. This study explored midwifery competency gaps in Kenya to recommend evidence-based policies, aiming to enhance maternal and neonatal health outcomes and inform future interventions.

**Methods:**

This study utilised a sequential explanatory study design which includes integrating quantitative and qualitative approaches of data collection, analysis, and synthesis. The quantitative phase involved a cross-sectional survey assessing midwives’ competence and confidence based on the International Confederation of Midwives (ICM) domains. 2019. The qualitative phase consisted of interviews with midwives and stakeholders to further explore and elucidate the quantitative findings. Sampling, interpretation, and reporting were integrated throughout the design. Data collection occurred in two distinct phases: an initial quantitative survey followed by qualitative interviews using a qualitative descriptive explanatory design with selected midwives and stakeholders. Qualitative data was coded and analyzed using Atlas ti 9 software. Thematic analysis, based on the Colaizzi framework, was conducted to capture emerging meanings while preserving the essence presented by the informants. Quantitative data were analyzed using SPSS, with descriptive statistics and statistical significance tested using Pearson’s Chi-square and Kruskal-Wallis tests. Both qualitative and quantitative data were analyzed separately and then connected to explain the results, ensuring triangulation and complementarity for comprehensive insights.

**Results:**

Direct-entry diploma midwives (KRM) and those working in tertiary hospitals reported higher competence and confidence (*P* = 0.019, *p* < 0.001, Kruskal-Wallis). KRMs were more confident in the ICM labour and birth domain (*p* = 0.017). Qualitative analysis revealed four themes: qualifications, enabling environment, work experience, and optimizing midwifery. Findings suggest that direct entry midwifery programs’ duration and education significantly impact midwives’ skill development. Mentorship and clinical supervision enhanced midwives’ confidence in labour and delivery domains. No statistical correlation was found between competence and work experience, but qualitative findings highlighted the importance of continuous professional development, clinical teaching, and supervision. An enabling working environment with appropriate resources, guidelines, and a multi-disciplinary team of obstetrics experts supported the high competence and confidence levels of midwives in tertiary care.

**Conclusion:**

This study identifies midwifery competency gaps in Kenya and recommends strengthening continuous professional development, clinical teaching, and supervision to improve maternal and newborn outcomes. It fills a knowledge gap by exploring midwives’ self-perceived competence and confidence levels in various domains of International Confederation of Midwives (ICM) competency through a sequential explanatory study design.

**Supplementary Information:**

The online version contains supplementary material available at 10.1186/s12913-025-13576-x.

## Background

The International Confederation of Midwives (ICM) recognises a midwife as a responsible and accountable professional who works in partnership with women to give the necessary support, care and advice during pregnancy, labour and the postpartum period, to be responsible for the conduct of births; and to provide care for the newborn and the infant [1].

This underscores the fact that midwives must have completed a midwifery education program that is based on the ICM Essential Competencies for Midwifery Practice and the framework of the ICM Global Standards for Midwifery Education that is recognised in the country where they are located and must demonstrate competency in the practice of midwifery [2].

These ICM essential competencies for midwifery practicee (i) general competencies, (ii) pre-pregnancy and antenatal care, (iii) care during labour and birth (iv) ongoing care of women and newborns; these competencies are defined as the minimum set of knowledge, skills, and professional behaviours required by an individual to use the designation of midwife when entering midwifery practice [3].

As prescribed by the World Health Organisation and ICM, to be competent practitioners, midwives must possess the requisite knowledge, skills, behaviours, and experience to provide evidence-based, human rights-based, quality, socio-culturally sensitive, and dignified care and to facilitate physiological processes during labour and delivery to ensure a clean and positive childbirth experience. In addition, they are expected to identify and manage or refer women or newborns with complications and must be able to perform all signal functions of emergency maternal and newborn care to optimise the health and well-being of women and newborns [4].

Skilled health personnel (SHP) are expected to have appropriate exposure to clinical experience to gain competence and develop confidence in providing maternal and neonatal care services [5–7]. However, in low- and middle-income countries (LMICs), there are inadequacies in midwifery education, including deficiencies in competency-based curricula, qualified tutors, strategies for learners’ assessment, and the appropriate infrastructure to support clinical teaching [8–11]. The lack of up-to-date theoretical knowledge and clinical practice skills in midwifery education may have profound implications for producing competent midwives who can provide the full range of services needed [12].

Midwifery education in Kenya offers two main pathways: a direct entry program and an integrated nurse-midwife program. These programs lead to various qualifications ranging from certificates to doctoral degrees. The different categories of midwives include Kenya Registered Midwifery (KRM), Kenya Registered Community Health Nursing (KRCHN), and Bachelor of Science in Nursing (BScN). While the KRCHN and BScN programs are combined, providing training in nursing, midwifery, mental health and community health skills, the KRM program focuses solely on direct entry midwifery training [13, 14].

The midwifery syllabus in Kenya recommends a minimum of 15 supervised births, which falls below the minimum of 40 recommended by the ICM [15]. Deviation from the ICM standards of practice could even be higher in Kenya since most of the health workforce working in the maternal and neonatal health (MNH) facilities have undergone the integrated nursing and midwifery program, which has a combined set of competencies in midwifery, community health, mental health, and general nursing for work across all health services. However, this broad focus results in fewer hours dedicated to midwifery training, which may limit the acquisition of specialized midwifery skills [16]. As a result, investments are needed to improve the quality of midwifery education to address sexual, reproductive, maternal, newborn, and adolescent health needs, with a focus on competency-based education, as emphasised by the State of the World Midwifery Report [17]. The World Health Organisation’s framework for strengthening the quality of midwifery education emphasises that such education is urgently needed to improve the quality of care, end preventable maternal and newborn mortality and stillbirths, and deliver the agenda of universal health coverage (UHC). To achieve this, it recommends clinical education and supervision during pre-service and in-service education to deliver education that provides the midwife with confidence to provide the full scope of midwifery care [18].

Recent investments in midwifery education to increase the production and deployment of midwives and expand access to facility birthing continue to influence good maternal outcomes in LMICs [19, 20]. This suggests that countries should invest and adapt their midwifery education to achieve the intended international competencies among midwives, which may improve access and quality of care.

The existing gap in midwifery education, highlighted by the State of the World Midwifery 2021 report, recommended that there is a need to review midwifery education to promote the acquisition of essential competencies for midwife practice [21] This study’s report highlights how qualitative findings on the experiences of midwives, and insights of stakeholders on competence and confidence, can explain the quantitative findings of self-perceived competence and confidence levels of midwives’ knowledge and skills, based on ICM components in Kenya. Findings on midwives’ competence and confidence have been published in a separate journal [22, 23].

Competence and confidence are essential concepts in midwifery practice and education. Competence refers to the minimum set of knowledge, skills, and professional behaviours required for midwives to effectively perform their duties, as defined by the International Confederation of Midwives [24–26], this competence enables midwives to provide safe and effective care before, during, and after childbirth [27].

Confidence, on the other hand, is the self-assurance that arises from an appreciation of one’s abilities and is closely linked to the acquisition of skills and knowledge as well as the practice environment [6, 28]. While confident midwives are often better equipped to make critical decisions that influence maternal and neonatal outcomes [29], it is important to note that confidence does not always equate to actual competence; individuals may feel confident without possessing the necessary skills, which can pose risks in professional settings [29].

Other factors that may influence confidence, include self-beliefs, contextual factors, and also societal influences. Thus, confidence is an essential factor that supports safe and professional performance. Hence, it is crucial to look at both confidence and competence in competency-based medical education [30].

The relationship between competence and confidence is significant but complex, as both are influenced by various factors, which may include hands-on experience in clinical settings and supportive learning environments [6, 30]. Studies have shown that perceived competence is often supported by actual knowledge and skills as well as the practice environment [6, 22].

However, challenges arise in measuring these constructs due to factors such as subjectivity in self-assessments, contextual variations affecting confidence levels, and cultural differences influencing perceptions of abilities [30].

Additionally, a study on midwifery education developed a self-assessment tool for educators to measure their confidence in competence, highlighting the need for valid measurement instruments [31]. However, measuring confidence and competence presents challenges such as subjectivity in self-assessments, contextual variations affecting confidence levels, and cultural differences impacting perceptions of abilities [30, 32, 33]. Overall, understanding the interplay between confidence and competence is crucial for enhancing educational outcomes and professional performance [33].

The reliance on self-assessment by midwives in practice to gauge their confidence in competence can result in response bias, where individuals may select responses that do not accurately reflect their true beliefs [30, 31].This is often due to apprehension about potential negative consequences associated with admitting low self-competence. Research indicates that many individuals experience anxiety when disclosing perceived inadequacies, leading to inflated self-assessments [6]. Such biases can compromise the validity of self-reported data, as midwives may feel compelled to portray themselves more favourably, ultimately affecting the accuracy of the evaluation process [34, 35]. When the midwives overestimate their competence, it may result in inadequate preparation for their students and negatively influence patient care [30].

Evidence from developing countries on the experiences of midwives and their confidence to practice and acquire competencies after initial education reports that midwives were confident in managing normal pregnancy, labour and delivery but felt inadequate in managing high-risk situations. In addition, clinical skills acquisition enhanced their confidence [6, 7, 36]. However, these studies were conducted utilising quantitative or qualitative approach methods and were therefore limited in their ability to explore or measure the findings as opposed to utilising a more robust, mixed methods approach, which may be able to more deeply understand findings by combining both qualitative and quantitative approaches [13, 20, 21].

The integration of quantitative and qualitative findings has the potential to generate a fuller understanding of the strengths and weaknesses of current midwifery education in Kenya. Additionally, the combined approach may lead to an improved approach to education and practice through the development of a midwifery professional framework that will enhance midwives’ competence and confidence in clinical practice, improving the quality of care to the woman and the community.

This study aims to understand how the qualitative findings on midwives’ experiences and stakeholders’ insights on competence and confidence can explain the quantitative findings of self-perceived competence and confidence.

## Methods

### Study design

This study is part of a series of studies assessing midwives’ self-perceived competence and confidence. The study utilised an explanatory, sequential design that involved connecting the quantitative and qualitative phases.

The quantitative phase of this study has been published [22, 23]. We used the results from the quantitative phase that assessed the self-perceived competence and confidence of midwives based on ICM domains to determine whom to sample to elaborate on the qualitative results. Additionally, self-perceived competence and confidence results guided the formulation of the interview protocol for the subsequent qualitative phase.

**Fig. 1:**
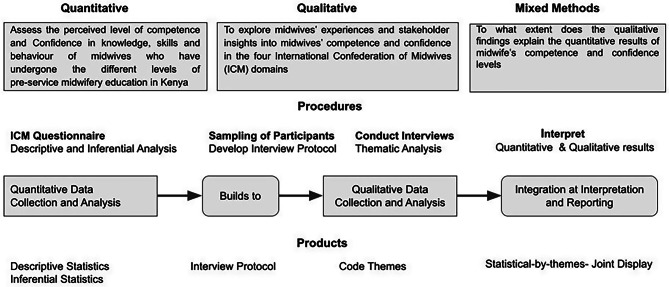
Explanatory sequential study design

By employing an explanatory sequential design, the study initially utilised quantitative methods to explore the levels of competence and confidence, followed by qualitative analysis to provide explanations. This approach enabled researchers to understand any discrepancy between quantitative results and qualitative findings [37–41], the overall study design is summarised in Fig. 1.

### Setting

The study setting was public healthcare facilities in Kenya. Kenya is in East Africa. It has a population of approximately 47 million and a growth rate of 2.4% annually. It is administratively divided into 47 counties, and the provision of health services is devolved to the county level [42, 43].

Healthcare in Kenya is offered at different levels: primary (dispensaries and health centres), secondary (sub-county and county referral hospitals), and tertiary (national referral hospitals) levels [44]. The study was conducted in sub-county, county, and referral facilities that offer comprehensive reproductive, maternal, and newborn child health (RMNCH) services. Private and faith-based facilities were excluded, as midwives may not always practice autonomously in these settings.

### Overview of methods for phase one

In this phase, the study population was the nurse-midwives who had undergone KRM, KRCHN, and BSCN programs, were active in clinical practice, and were deployed in primary, secondary, or tertiary public hospitals. The midwives deployed at private and faith-based institutions and training institutions were excluded from the study. Most of the private sector had not employed the three cadres of nurses under study; they lacked the appropriate skills mix. In addition, their scope of practice in midwifery is restricted.

The study employed an observational cross-sectional design to assess midwives’ self-perceived competence and confidence using the adapted ICM self-assessment tool. The tool, contextualized for Kenya by a panel of nine experts, underwent content validation and a pilot study with 50 participants, achieving a Cronbach’s alpha of 0.87. The study sampled 576 midwives from public facilities using stratified multi-stage sampling, ensuring representation across qualifications (KRM, KRCHN, and BScN) and facility levels (primary, secondary, and tertiary). Data collection occurred between December 2019 and March 2020, using paper-based questionnaires later digitized in ONA software. Research assistants, recruited from non-study facilities to minimize bias, conducted data collection, lasting 40–60 min per participant. Of the invited participants, all consented; reasons for any potential refusals were not reported. Data were analyzed using SPSS, descriptive statistics and statistical significance were tested using Pearson’s chi-square and Kruskal-Wallis tests.

### Overview of methods for phase two

In this phase, the study utilised a qualitative descriptive approach to explore midwives’ competence and confidence experiences during practice. The study population included two groups: nurse-midwives drawn from Phase One and stakeholders, comprising chief nursing officers and chief executive officers of the counties where data were collected in Phase One. During the quantitative phase, participants were informed about a potential follow-up qualitative phase, with interview protocols derived from the quantitative findings.

Twelve midwives were purposively selected based on low, moderate, and high competence and confidence levels identified in phase one. Participants were from public sub-county, county, and tertiary hospitals, excluding private and faith-based facilities due to limited autonomy. Nine chief nursing officers (CNOs) from selected counties and one chief executive officer (CEO) from a national referral hospital were also included.

Data was collected through the Interview guides that were developed based on key findings from phase one of the study (quantitative phase). Qualitative understanding and experiences of the midwives’ level of preparedness (competence and confidence) to offer midwifery services were explored. Perceptions of stakeholders and midwives’ experiences about their knowledge and skills in the four ICM domains while discharging their midwifery duties were also explored.

Two experienced research assistants, trained in qualitative data collection, conducted the interviews in English using a blended approach of face-to-face and virtual methods, depending on COVID-19 restrictions. Interviews were conducted at locations convenient for participants to ensure privacy and comfort.

Of the invited participants, eight midwives and seven stakeholders participated. Refusal reasons included scheduling conflicts and personal commitments. Data saturation was achieved at this point.

Data was coded and analysed using the Atlas ti 9 software. Thematic analysis using the Colaizzi framework was undertaken to describe the emerging meanings without losing the essence presented by the informant [45, 46].

### Integration at the design stage, interpretation and reporting level

Integration was embedded in the study design from the conceptualization stage, ensuring coherence between phases. The qualitative phase (Phase Two) was designed to build on the findings of the quantitative phase (Phase One) through data collection and analysis. Integration was further operationalized at the methods stage by linking the quantitative dataset to the qualitative sampling process. Additionally, the two phases were integrated during the interpretation of findings, with qualitative insights providing context and explanation for the quantitative results.

Joint displays were used to interpret the combined quantitative and qualitative data The statistics-by-theme joint displays compared the quantitative results derived from statistical results and qualitative findings displayed as the emerging themes [47]. Quantitative and qualitative data were analysed separately; the results and findings from both phases were connected based on statistical results in the quantitative phase and the emerging themes of the qualitative phase. The connected results were interpreted to answer the mixed methods research question of how qualitative findings explain quantitative results [48].

Regarding fit, the qualitative findings confirmed and contextualised the quantitative findings; this occurs when the findings from both types of data confirm the results of the other combining both methods facilitated triangulation and complementarity; triangulation was used to cross-check results within the study [49–51].

## Results

 Through the mixed methods integration approach, we identified explanations for variations in competence and confidence levels among the three categories of midwives. Our analysis identified four key themes: qualifications, creating an enabling environment, clinical practice experience, and the importance of optimising midwifery. The study findings are presented in Table 1, which presents a joint display of mixed methods integration that showcases a combination of results, qualitative insights, and explanations linking them together [50, 52].

### Qualifications

Direct entry midwives (KRM) demonstrated higher knowledge levels (81.8%) compared to integrated nurse midwives (KRCHN 75.3% and BSCN 76.9%; p = 0.019). Moreover, KRMs exhibited better skill proficiency (93.2%) than integrated nurse midwives (KRCHN 81.7% and BSCN 79.5%; p = 0.016). Qualitative findings highlight the significant impact of duration and education in direct-entry midwifery programs on midwives’ skill development. Increased exposure to midwifery-specific subjects and practical training enhances proficiency in midwifery skills. While direct entry midwifery education enhances competency, further education is necessary to expand knowledge and specialise in midwifery. In contrast, KRCHNs and BScNs who underwent nurse midwifery blended programs may not attain the same level of expertise in midwifery as direct entry midwifery education.

### Enabling environment

 Quantitative findings indicated that midwives exhibited more competence and confidence in midwifery at tertiary institutions compared to secondary (county) and primary-level (sub-county) settings (p < 0.001) [22, 23].

Qualitative findings supported this, attributing midwives’ high competence and confidence levels at tertiary care to an enabling working environment with appropriate resources, guidelines, and a multi-disciplinary team of obstetrics experts. To foster midwives’ confidence and competence, it is essential to establish multidisciplinary support and standardised practices.

### Clinical practice experience

While no statistical correlation was found between competence and work experience, qualitative findings emphasised the importance of years of experience, continuous professional development, clinical teaching, and supervision in fostering midwives’ competence and confidence. The extensive work experience of KRM participants is a possible explanation for significantly boosting their confidence and competence across midwifery domains. Supervision’s absence could impede skill acquisition during both educational and practical phases, indicating a gap in midwifery training. Additionally, gaining hands-on experience over the years and engaging in professional development within a supportive work environment potentially contribute to improving clinical practice experience and the overall competence of midwives.

### Optimising midwifery

There were variations in competence and confidence levels across the four ICM domains: general competence, pre-pregnancy and antenatal care, labour and birth, and ongoing care for the woman and newborn. Midwives exhibited higher confidence levels in ongoing care (81.2%) and labour and childbirth (82.0%). KRMs showed greater confidence than KRCHNs and BScNs in the labour and birth domain (*p* = 0.017) [23]. Qualitative insights highlighted the significant role of mentorship and clinical supervision in enhancing midwives’ confidence and abilities in care provision. Discrepancies in confidence and competence among midwives may be attributed to the availability of resources and adherence to evidence-based protocols and clinical guidelines, particularly during labour and delivery. To enhance midwifery competence, optimising midwifery practices with a focus on practice improvement is crucial for strengthening competence and confidence levels, were the possible reasons provided.

**Table 1:** Joint display of integrated findings

Theme	Quantitative Results:	Qualitative findings and Illustrative quotes	Meta-inference
Qualifications	**Quantitative Results**: Direct-entry diploma midwives (KRM) showed significantly greater competence levels compared to integrated Nurse-Midwife programs	The qualification categories influence the midwives’ competence levels. KRM exhibit superior knowledge and safe skill performance due to their extensive midwifery education. *“… If we have people who have done midwifery alone*,* they have difference [they are] more competent because all they do is about midwifery.” Midwife 6* *“We need to take our midwifery back to those days – KRM-qualified midwives*,* where a midwife could assess and diagnose a woman in labour and know if she could give birth comfortably … midwives could even conduct breech delivery comfortably. I think we need to take our midwifery back there” [where there was no integration of nursing and midwifery qualifications] (Stakeholder 3)*	The qualitative findings indicate that the duration and education provided in direct-entry midwifery programs significantly impact midwives’ skill development. Extended exposure to midwifery-specific subjects and practical training enhances midwifery skills proficiency. Direct entry to midwifery education improves competency, but further education is deemed necessary to broaden knowledge and specialize in midwifery. In contrast, KRCHNs and BScNs, who integrate nurse midwifery content, may not achieve the same level of expertise in midwifery as direct entry midwifery education.
Enabling environment	Midwives working at tertiary hospitals reported higher confidence levels than county and sub-county hospitals	The competence and confidence of midwives working in tertiary hospitals emerged because of exposure to more complicated cases, appropriate health infrastructure, resources, and multidisciplinary support resources. *“The other thing that boosts their confidence is know that even if the midwives are there (in tertiary hospitals)*,* there are also the doctors*,* the consultants are also there*,* we have several consultants’ gynaecologists*,* and also the realization that if all goes wrong*,* theatre is within reach; that also boosts your confidence*,* that you do not have to struggle…” (Midwife 22)* *“… now a midwife who is in a level five [Tertiary] could have better exposure than*,* of course*,* the one who is in level four (county) and three (sub-county)*,* because the one in level three*,* when a mother develops irregular foetal heart rate*,* the next thing is you prefer. But the one who is in level five has more experience and resources*,* maybe even more competence…” Stakeholder 5*	Qualitative findings ***confirmed*** that an enabling working environment equipped with appropriate resources and guidelines and a multi-disciplinary team of experts in obstetrics to support the provision of care surfaced as possible **explanations** for the high competence and confidence levels of Midwives deployed at the tertiary level of care.
Clinical practice experience	There was no significant association between competence, confidence levels and work experience. However, the KRM respondents were significantly older and had more years of experience.	Hands-on experience and cumulative years of experience in practice were highlighted as essential to build the midwife’s confidence and enhance competence in practice. *“…So*,* the level of confidence goes with the number of years the person has taken in the department.” Stakeholder 2* “*To me*,* school is just getting the knowledge. But the practical skills start when you start working*,* and the new midwives [novices] should be supervised.” Stakeholder 6* Further, the clinical experience emerged as being enhanced by supervision. It was also highlighted that there was a gap in the clinical supervision of the midwives. “*If you really want a lot of competence*,* you really must press on so much on the practicum and a lot of follow-ups*,* a lot of supervision which I feel is lacking.” Stakeholder 6*	While no statistical correlation was found between competence and work experience, qualitative findings emphasized the importance of years of experience, continuous professional development, clinical teaching, and supervision in fostering midwives’ competence and confidence. The extensive work experience of KRM participants significantly boosted their confidence and competence across midwifery domains. The absence of supervision could potentially impede skill acquisition during both educational and practical phases, indicating a potential gap in midwifery training.
Optimizing Midwifery	There were significant differences in midwives’ confidence in the ICM domains (labour and birth)	Adequate midwifery education enabled KRM midwives to be perceived as having high competence in the labour and birth ICM domains of competence, attributed to being deployed to work in labour and delivery units. *“What I can say about education*,* you know it was a broad education*,* …. on everything*,* but now when you go to specific midwifery*,* you find that you are confident in childbirth and you have that competence. I can say the KRM is not like the KRCHN education; it is so broad. KRM manage our labour ward. When there is specialization*,* you can be confident in that specialization ”Midwife 3.* “*Generally*,* I [KRM Midwife] think I have a high confidence in midwifery*,* given that sometimes you are alone with a normal client or a mother and you can assess the mother from the first stage to the third stage*,* there are guidelines*,* I mean SOPs [Standard operating procedures]*,* and you can be able to detect if the mother has any complications” Midwife 10.*	Qualitative findings provided insights into how mentorship and clinical supervision significantly contributed to enhancing their confidence and abilities in providing care. The differences observed could be attributed to the availability of resources utilized in various domains and the implementation of evidence-based protocols and clinical guidelines, which are mostly followed during labour and delivery.

## Discussion

This study found that direct-entry diploma midwives (KRM) and those in tertiary hospitals reported higher competence and confidence. KRMs were more confident in the ICM labour and birth domain. Qualitative analysis identified four themes: qualifications, enabling environment, work experience, and optimizing midwifery. Direct entry programs’ duration and education significantly impacted skill development, while mentorship and clinical supervision enhanced confidence in labour and delivery. Continuous professional development, clinical teaching, and an enabling environment with resources and a multi-disciplinary team supported high competence and confidence. This sequential explanatory methods integrated quantitative and qualitative findings at all study levels, providing a comprehensive understanding of midwives’ self-perceived confidence and competence in the four ICM domains [47, 48].

Overall, there were significant differences in the confidence and competence levels among the different midwives’ cadres. These differences were seen in the different levels of the facility and the different ICM domains [21, 22].

### Qualifications

Direct-entry midwives were seen as more knowledgeable and skilled, particularly when their education emphasized independent midwifery practice. Their competence and confidence levels were influenced by clinical experience and supervision. Midwives demonstrated greater competence and confidence in labour and childbirth. Higher levels of care correspond to higher competence and confidence.

Our mixed methods integration reveals that direct-entry midwives report higher confidence and competence due to their exposure to actual midwifery practice. These results align with previous studies that also found higher levels of confidence and competence among direct-entry midwives [22, 23, 53–55].

Also, the higher confidence and competence observed among direct-entry midwives can be attributed to an educational curriculum that exposes them to a wide range of midwifery experiences over an extended period. This exposure promotes competence and confidence when compared to integrated nurse-midwifery programs [55, 56].

The extended and diverse midwifery experiences in KRM education contribute to their increased confidence and competence. To enhance midwife competence and confidence, reviewing midwifery program curricula and structures is crucial, focusing on ICM’s four domains. Providing adequate clinical practice supervision and continuous professional development opportunities for in-service midwives is also essential [55–58].

### Clinical practice experience

Most of the midwives in Kenya have undergone the integrated nurse-midwifery programme [14, 59] and direct-entry midwives’ high confidence and competence result from clinical practice with a wide range of midwifery experiences over an extended period. In contrast, integrated nurse-midwifery programs may not provide the same level of competence and confidence, indicating a need for continuous professional development and clinical teaching to enhance their competence and confidence. To improve the integrated nurse-midwife program, a competency-based learning approach should be implemented, focusing on practical skills through clinical supervision to achieve ICM essential competencies [60].

Additionally, increasing practical exposure during education and after graduation can enhance midwives’ confidence in practice. Intervention studies support the importance of relevant clinical placement and clinical supervision in enhancing midwives’ knowledge and hands-on practice practice [61–64].

### Enabling environment

This study found that midwives from tertiary referral hospitals have higher confidence and competence in their knowledge and clinical skills than those from county and sub-county hospitals. Factors contributing to this difference include sufficient exposure to learning, a variety of patients, adequate resources, and a specialist multi-disciplinary team at tertiary facilities [44, 58].

Enhancing midwives’ competence and confidence is supported by an enabling environment, a positive workplace culture and multi-disciplinary clinical teaching [65–68].

Given the stark differences in self-perceived knowledge and skills between midwives from tertiary referral hospitals and those from county/sub-county hospitals, examining the quality of midwifery care these facilities provide is necessary. An estimated 15% of pregnant women will develop a complication during pregnancy, childbirth, or the puerperium, which will require EMOC-trained, skilled health personnel [69]. The findings indicate that midwives working at county and sub-county levels felt they had lower confidence and competence according to ICM standards. These midwives manage most pregnancies, often in lower-level facilities that form the majority of healthcare centres in the country. This situation is alarming, particularly given the first confidential enquiry into maternal deaths in Kenya, which reported that 9 out of 10 maternal deaths were associated with substandard care [70, 71].

The workplace environment and practice can influence midwives’ scope of practice, which varies according to facility levels and accessibility of experts [71, 72]. To improve confidence and competence in midwifery practice and maternal and newborn health outcomes, investment is needed to build capacity in terms of capabilities, motivation, and a conducive learning and supportive environment for midwives at lower-level facilities [67, 73, 74].

### Optimising midwifery

This study found that midwives in Kenya have good knowledge and skills in labour and birth but less confidence and competence in other domains (general competence, pre-pregnancy and antenatal care and ongoing care of the woman and newborn), which is concerning as half of maternal deaths occur during the intrapartum and postpartum period [72, 75–77].

Other studies have also reported that there is less confidence among midwives in postnatal and newborn care [54, 65], similar findings show that technical preparedness may not ensure good quality services and client satisfaction in antenatal and delivery care [78, 79]. To improve midwifery competence and confidence, investment is needed in midwifery education and ensuring that midwives have the requisite competencies in essential emergency obstetrics and newborn care, as well as contextually and culturally appropriate competencies [18, 80]. The current midwifery program should be reviewed according to ICM competencies, and faculty education and clinical practice supervision are strategies to enhance midwives’ competence and confidence [22, 55, 67, 81].

This article contributes to filling the knowledge gap on the competence and confidence of midwives in Kenya, using the mixed methods approach, and produces recommendations for enhancing midwives’ competence and confidence. The focus of this study was the perceived competence and confidence level of midwives. Thus, researchers need to measure midwives’ actual competence and evaluate the pre-service programmes.

### The implications for practice

#### Qualifications

Direct-entry midwives show greater confidence and competence in ICM domains compared to integrated nurse-midwife programs, suggesting potential care quality issues. To meet UHC targets, midwives should be trained in direct-entry programs, and integrated pathways reviewed for ICM standards compliance. Developing a standardized competency framework and enhancing midwives’ skills through continuous professional development, supervision, and mentorship is crucial for improving maternal and newborn outcomes.

#### Optimising midwifery

Midwives reported higher competence and confidence in labour and birth domains, impacting the continuum of care. Support through supervision, mentorship, and comprehensive curriculum covering all ICM domains, including enhanced clinical assessment, is essential for optimal midwifery practice.

#### Clinical practice experience

Inadequate clinical practice experience among midwives can lead to poor competence and negatively impact neonatal and maternal outcomes. Enhancing clinical learning, continuous professional development, and establishing county-level referral facilities with shared clinical specialists are crucial. Implementing outreach, in-reach programs, and exchange visits by skilled healthcare personnel is essential.

#### Enabling environment

Working in an enabling environment significantly influenced midwives’ competence and confidence. Midwives at tertiary facilities reported higher competence due to exposure to quality care, expert teams, and resources. However, midwives at county and sub-county facilities reported lower competence, potentially leading to sub-optimal care. To support quality service delivery, it is crucial to provide resources for infrastructure, adequate staffing, and skills mix. Developing a rotational schedule for midwives and establishing dispensaries and community service units are recommended for continuous quality improvement across all health centers.

## Conclusions

This studyfound that current midwifery education and education in Kenya have mixed results, with direct-entry trained midwives perceived to be more confident and competent in their knowledge and skills than the integrated nurse-midwives. Health system challenges exist between tertiary facilities and county and sub-county facilities, as tertiary facilities provide a better learning environment than facilities at the county level. Midwives should be trained in direct-entry midwifery programmes that meet ICM education standards; the current integrated nurse-midwifery programmes should be reviewed to standardize the competencies for improved maternal and newborn outcomes. Therefore, policymakers and health planners should place significant emphasis on addressing the identified midwifery competency gaps in Kenya. It is recommended to strengthen continuous professional development, clinical teaching, and supervision to enhance maternal and newborn outcomes.

## Supplementary Information

Below is the link to the electronic supplementary material.

Supplementary Material 1

## Data Availability

The datasets generated and/or analysed during the current study are not publicly available due confidentiality of the data but are available from the corresponding author upon reasonable request.

## Abbreviations

BSCN: Bachelor of Science in Nursing
CEO: Chief Executive Officer
ICM: International Confederation of Midwives
KRCHN: Kenya Registered Community Health Nurse
KRM: Kenya Registered Midwife
