# Supplementary material for: Midwives’ competence and confidence in Kenya: a sequential explanatory study design
Source: BMC Health Serv Res. 2026 Jan 13;26:208. doi: 10.1186/s12913-025-13576-x (PMC12888656; doi:10.1186/s12913-025-13576-x)
Supplement: Supplementary file 1 — Supplementary Material 1 [file 12913_2025_13576_MOESM1_ESM.pdf]

Data collection tool adopted from ICM

A. Demographic Information

1.1 Please select all your qualifications from the options below :(Please tick more than one ifapplicable)

KRM KRCHN BSCN

1.2 List any other qualifications not captured in the above question

Post basic diploma Master's degree PhD. Others, Please Specify

1.3 Indicate your professional registration with NCK. Indicate all that apply.

Nurses. Midwives. Bachelors. Higher Diploma Master's degree

1.4 List any NCK registrations not selected in 1.3

1.5 What is your age in years?

1.6 Please indicate your gender?

1.7 How many years of Work experience do you have as a Midwife/ Nurse Midwife

1.8 What is your area of expertise

Neonatology. Midwife Reproductive HealthOthers please specify

1.9 Please indicate your facility type. Level 6 Level 5 level 4 level 3

B. Monitoring Midwifery Competencies Self-Assessment Tool

ICM Essential Competencies	COMPETENCE						CONFIDENCE				
	Knowledge (e.g. CPDs, guidelines)			Skill and Behaviour			Self-rating of confidence in current knowledge OR				
Knowledge, Skill, and Behaviour	Not updated my knowledge(>3years)	Updated my knowledge (>2-3 years)	Current in my knowledge (within 1year)	Not performed this skill safely withinthe past year	Performed this skill safely oncetime within the past year	Performed this skill safely on morethan one occasion within the past	Not at all		somewhat		Very confident
							1	2	3	4	5
**CATEGORY 1; GENERAL COMPETENCY**											
**COMPETENCY 1.1: Assume responsibility for own decisions and actions as an autonomous**											
**a) KNOWLEDGE**											
i. Principles of accountability and											
ii. Principles and conceptsof											
iii. Personal beliefs andtheir influence on practice											
iv. Knowledge of evidence-based											
**b) SKILLS AND BEHAVIORS**											
i. Demonstrate behaviour that upholds the public trust in the											
ii. Participate in self-evaluation, peer reviewand other quality improvement											
iii. Balance the responsibility of the midwife to provide best care with the autonomy of the woman tomake											

ICM Essential Competencies	COMPETENCE						CONFIDENCE				
	Knowledge (e.g. CPDs, guidelines)			Skill and Behaviour			Self-rating of confidence in current knowledge OR				
Knowledge, Skill, and Behaviour	Not updated my knowledge(>3years)	Updated my knowledge (>2-3 years)	Current in my knowledge (within 1year)	Not performed this skill safely withinthe past year	Performed this skill safely oncetime within the past year	Performed this skill safely on morethan one occasion within the past	Not at all		somewhat		Very confident
							1	2	3	4	5
iv. Explain the midwife's rolein providing care that is based on relevant law, ethics, and evidence											
**COMPETENCY 1.2 Assume responsibility for self-care and self-development as a midwife**											
**a) KNOWLEDGE**											
i. Strategies for managing personal safety particularlywithin the facility or											
**b) SKILLS AND BEHAVIORS**											
i. Display skills in management of self in relation to time management, uncertainty,change and coping with stress											
ii. Assume responsibility for personal safety in various practice											
iii. Maintain up-to-date skills and knowledge concerningprotocols, guidelines, and safe											
**iv.** Remain current in practiceby participating in continuing professional education											

ICM Essential Competencies	COMPETENCE						CONFIDENCE				
	Knowledge (e.g. CPDs, guidelines)			Skill and Behaviour			Self-rating of confidence in current knowledge OR				
Knowledge, Skill, and Behaviour	Not updated my knowledge(>3years)	Updated my knowledge (>2-3 years)	Current in my knowledge (within 1year)	Not performed this skill safely withinthe past year	Performed this skill safely oncetime within the past year	Performed this skill safely on morethan one occasion within the past	Not at all		somewhat		Very confident
							1	2	3	4	5
participating in learningopportunities that applyevidence to practice improving care such as mortality reviews or policy reviews.)											
v. Identify and address limitations in personalskill, knowledge, or experience											
vi. Promote the profession ofmidwifery, including participation in professional organizationsat the local and national level											
**COMPETENCY 1.3 Appropriately delegate aspects of care and provide supervision**											
**a) KNOWLEDGE**											
i. Policies and regulationrelated to											
ii. Use supportive strategiesto supervise											
iii. Role of midwives as preceptors, mentors, androle models											
**b) SKILLS AND BEHAVIORS**											

ICM Essential Competencies	COMPETENCE						CONFIDENCE				
	Knowledge (e.g. CPDs, guidelines)			Skill and Behaviour			Self-rating of confidence in current knowledge OR				
Knowledge, Skill, and Behaviour	Not updated my knowledge(>3years)	Updated my knowledge (>2-3 years)	Current in my knowledge (within 1year)	Not performed this skill safely withinthe past year	Performed this skill safely oncetime within the past year	Performed this skill safely on morethan one occasion within the past	Not at all		somewhat		Very confident
							1	2	3	4	5
i. Provide supervision toensure that practice is aligned with evidence-based clinical											
ii. Support the profession' s growth through participation in midwiferyeducation in the roles of clinical preceptor, mentor,											
**COMPETENCY 1.4 Use research to inform practice**											
**a) KNOWLEDGE**											
i. Principles of research andevidence-based practice											
ii. Epidemiologic concepts relevant to maternal andinfant health											
**b) SKILLS AND BEHAVIORS**											
i. Discuss research findings with women, families andcolleagues											
ii. Support research in midwifery by participatingin the conduct of research											
**COMPETENCY 1.5. Uphold fundamental human rights of individuals when providing**											

ICM Essential Competencies	COMPETENCE						CONFIDENCE				
	Knowledge (e.g. CPDs, guidelines)			Skill and Behaviour			Self-rating of confidence in current knowledge OR				
Knowledge, Skill, and Behaviour	Not updated my knowledge(>3years)	Updated my knowledge (>2-3 years)	Current in my knowledge (within 1year)	Not performed this skill safely withinthe past year	Performed this skill safely oncetime within the past year	Performed this skill safely on morethan one occasion within the past	Not at all		somewhat		Very confident
							1	2	3	4	5
**a) KNOWLEDGE**											
i. Laws and/or codes thatprotect human											
ii. Sexual, reproductive health rights of womenand girls											
iii. Development of genderidentity and sexual orientation											
iv. Principles of ethics andHuman Rights within midwifery											
**b) SKILLS AND BEHAVIORS**											
i. Provide information to women about their sexualand reproductive health											
ii. Inform women about thescope of midwifery practice and women' s											
iii. Provide information and support to individuals in complex situations where there are competing ethical											
iv. Practice in accordance with philosophy and codeof ethics of the											

ICM Essential Competencies	COMPETENCE						CONFIDENCE				
	Knowledge (e.g. CPDs, guidelines)			Skill and Behaviour			Self-rating of confidence in current knowledge OR				
Knowledge, Skill, and Behaviour	Not updated my knowledge(>3years)	Updated my knowledge (>2-3 years)	Current in my knowledge (within 1year)	Not performed this skill safely withinthe past year	Performed this skill safely oncetime within the past year	Performed this skill safely on morethan one occasion within the past	Not at all		somewhat		Very confident
							1	2	3	4	5
national standards for health professionals											
v. Provide gender sensitivecare											
**COMPETENCY 1.6 Adhere to jurisdictional laws, regulatory requirements, and codes of conduct formidwifery practice**											
**a) KNOWLEDGE**											
i. Explain the laws and regulations of the jurisdiction regarding											
ii. National/state/local community standards ofmidwifery practice											
iii. Ethical principles											
iv. ICM and other midwiferyphilosophies, values, codes of ethics											
**b) SKILLS AND BEHAVIORS**											
i. Practice according to legalrequirements and ethical											
ii. Meet requirements for maintenance of midwiferyregistration											
iii. Protect confidentiality of oral information and written records about careof women and											

ICM Essential Competencies	COMPETENCE						CONFIDENCE				
	Knowledge (e.g. CPDs, guidelines)			Skill and Behaviour			Self-rating of confidence in current knowledge OR				
Knowledge, Skill, and Behaviour	Not updated my knowledge(>3years)	Updated my knowledge (>2-3 years)	Current in my knowledge (within 1year)	Not performed this skill safely withinthe past year	Performed this skill safely oncetime within the past year	Performed this skill safely on morethan one occasion within the past	Not at all		somewhat		Very confident
							1	2	3	4	5
iv. Maintain records of care in the manner required by thehealth authority											
v. Comply with all local reporting regulations for birth and death											
vi. Recognize violations oflaws, regulations, and ethical codes and take appropriate											
vii. Report and document incidents and adverse outcomes as required while											
**COMPETENCY 1.7 Facilitate women and families to make individual choices about care**											
**a) KNOWLEDGE**											
i. Cultural norms and practices surrounding sexuality, sexual practices,marriage, the childbearingcontinuum,											
ii. Principles of empowerme											
iii. Methods of conveyinghealth information to individuals, groups,											
**b) SKILLS AND BEHAVIORS**											

ICM Essential Competencies	COMPETENCE						CONFIDENCE				
	Knowledge (e.g. CPDs, guidelines)			Skill and Behaviour			Self-rating of confidence in current knowledge OR				
Knowledge, Skill, and Behaviour	Not updated my knowledge(>3years)	Updated my knowledge (>2-3 years)	Current in my knowledge (within 1year)	Not performed this skill safely withinthe past year	Performed this skill safely oncetime within the past year	Performed this skill safely on morethan one occasion within the past	Not at all		somewhat		Very confident
							1	2	3	4	5
i. Advocate for and supportwomen to be the central decision makers in their care											
ii. Assist women to identify their needs, knowledge, skills, feelings, and preferences throughout											
iii. Provide information andanticipatory guidance about sexual and reproductive health to assist women's											
iv. Collaborate with women in developing a comprehensive plan of care that respects her preferences and											
**COMPETENCY 1.8 Demonstrate effective interpersonal communication with women and families health care teams, and community groups**											
**a) KNOWLEDGE**											
i. Role and responsibilitiesof midwives and other maternal – infant											
ii. Principles of effective											

ICM Essential Competencies	COMPETENCE						CONFIDENCE				
	Knowledge (e.g. CPDs, guidelines)			Skill and Behaviour			Self-rating of confidence in current				
Knowledge, Skill, and Behaviour	Not updated my knowledge(>3 years)	Updated my knowledge (>2-3 years)	Current in my knowledge (within 1year)	Not performed this skill safely withinthe past year	Performed this skill safely oncetime within the past year	Performed this skill safely on morethan one occasion within the past	Not at all		somewhat		Very confident
							1	2	3	4	5
iii. Principles of effectivelyworking in health care teams											
iv. Cultural practices andbeliefs related to childbearing and reproductive health											
v. Principles of communication in crisis situations, e.g., grief and											
**b) SKILLS AND BEHAVIORS**											
i. Listen to others in an unbiased and empatheticmanner											
ii. Respect one others' pointof view											
iii. Promote the expression ofdiverse opinions and perspectives											
iv. Use the preferred languageof the woman or an interpreter to											
v. Establish ethical and culturally appropriate boundaries between professional and non-professional											

ICM Essential Competencies	COMPETENCE						CONFIDENCE				
	Knowledge (e.g. CPDs, guidelines)			Skill and Behaviour			Self-rating of confidence in current knowledge OR				
Knowledge, Skill, and Behaviour	Not updated my knowledge(>3years)	Updated my knowledge (>2-3 years)	Current in my knowledge (within 1year)	Not performed this skill safely withinthe past year	Performed this skill safely oncetime within the past year	Performed this skill safely on morethan one occasion within the past	Not at all		somewhat		Very confident
							1	2	3	4	5
vi. Demonstrate cultural sensitivity to women, families, and											
vii. Demonstrate sensitivity and empathy for bereavedwomen and family members											
viii. Facilitate teamwork and inter-professional care with other care providers(including students) and community											
ix. Establish and maintain collaborative relationshipswith individuals, agencies, institutions that are part											
x. Convey information accurately and clearly andrespond to the needs of individuals											
**COMPETENCY 1.9 Facilitate normal birth processes in institutional and community settings including women's homes**											
**a) KNOWLEDGE**											
i. Normal biologic, psychologic, social, and cultural aspects of reproduction and early											

ICM Essential Competencies	COMPETENCE						CONFIDENCE				
	Knowledge (e.g. CPDs, guidelines)			Skill and Behaviour			Self-rating of confidence in current knowledge OR				
Knowledge, Skill, and Behaviour	Not updated my knowledge(>3years)	Updated my knowledge (>2-3 years)	Current in my knowledge (within 1year)	Not performed this skill safely withinthe past year	Performed this skill safely oncetime within the past year	Performed this skill safely on morethan one occasion within the past	Not at all		somewhat		Very confident
							1	2	3	4	5
ii. Practices that facilitate andthose that interfere with normal processes											
iii. Policies and protocols about care of women in institutional and											
iv. Availability of resourcesin various											
v. Community views about and utilization of health care facilities and											
**b) SKILLS AND BEHAVIORS**											
i. Promote policies and a work culture that valuesnormal birth											
ii. Utilize human and clinicalcare resources to provide personalized care for women and											
iii. Provide continuity of careby midwives known to woman											
**COMPETENCY 1.10 Assess the health status, screen for health risks, and promote general healthand well-being of women and infants**											
**a) KNOWLEDGE**											
i. Health needs of womenrelated to											

ICM Essential Competencies	COMPETENCE						CONFIDENCE				
	Knowledge (e.g. CPDs, guidelines)			Skill and Behaviour			Self-rating of confidence in current knowledge OR				
Knowledge, Skill, and Behaviour	Not updated my knowledge(>3years)	Updated my knowledge (>2-3 years)	Current in my knowledge (within 1year)	Not performed this skill safely withinthe past year	Performed this skill safely oncetime within the past year	Performed this skill safely on morethan one occasion within the past	Not at all		somewhat		Very confident
							1	2	3	4	5
ii. Health conditions thatpose risks during reproduction											
iii. Health needs of infantsand common											
**b) SKILLS AND BEHAVIORS**											
i. Conduct a comprehensive assessment of sexual											
ii. Assess risk factors and at-risk behaviour											
iii. Order, perform, and interpret laboratory and/orimaging											
iv. Exhibit critical thinkingand clinical reasoning informed by evidence when promoting health and											
v. Provide health informationand advice tailored to individual circumstances of women and their											
vi. Collaborate with women to develop and implementa plan of											
**COMPETENCY 1.11 Prevent and treat common health problems related to reproduction and earlylife**											
**a) KNOWLEDGE**											

ICM Essential Competencies	COMPETENCE						CONFIDENCE				
	Knowledge (e.g. CPDs, guidelines)			Skill and Behaviour			Self-rating of confidence in current knowledge OR				
**Knowledge, Skill, and Behaviour**	Not updated my knowledge(>3years)	Updated my knowledge (>2-3 years)	Current in my knowledge (within 1year)	Not performed this skill safely withinthe past year	Performed this skill safely oncetime within the past year	Performed this skill safely on morethan one occasion within the past	Not at all		somewhat		Very confident
							1	2	3	4	5
i. Common health problemsrelated to sexuality and											
ii. Common health problems and deviations from normal											
iii. Treatment of commonhealth problems in Women, Children											
iv. Strategies to prevent and control the acquisition and transmission of environmental and											
**b) SKILLS AND BEHAVIORS**											
i. Maintain/promote safe andhygienic conditions for women and infants											
ii. Use universal precautions											
iii. Provide options to womenfor coping with and treating common health problems											
iv. Use technology and interventions appropriatelyto promote health and prevent secondary											

ICM Essential Competencies	COMPETENCE						CONFIDENCE				
	Knowledge (e.g. CPDs, guidelines)			Skill and Behaviour							
Knowledge, Skill, and Behaviour	Not updated my knowledge(>3years)	Updated my knowledge (>2-3 years)	Current in my knowledge (within 1year)	Not performed this skill safely withinthe past year	Performed this skill safely oncetime within the past year	Performed this skill safely on morethan one occasion within the past	Self-rating of confidence in current knowledge OR				
							Not at all		somewhat		Very confident
							1	2	3	4	5
v. Recognize when consultation or referral is indicated for managing identified health problems, including consultation											
vi. Include woman in decision-making about referral to other providersand services											
**COMPENTENCY 1.12 Recognize conditions outside midwifery scope of practice and referappropriately**											
**a) KNOWLEDGE**											
i. Complications/pathologic conditions related to											
ii. Limits of midwifery scopeof practice and own experience											
iii. Available referral systemsto access medical and other personnel to manage											
iv. Community/facility plansand protocols for accessing resources in timely manner											
**b) SKILLS AND BEHAVIORS**											
i. Maintain up-to-date knowledge, skills,											

ICM Essential Competencies	COMPETENCE						CONFIDENCE				
	Knowledge (e.g. CPDs, guidelines)			Skill and Behaviour			Self-rating of confidence in current knowledge OR				
Knowledge, Skill, and Behaviour	Not updated my knowledge(>3years)	Updated my knowledge (>2-3 years)	Current in my knowledge (within 1year)	Not performed this skill safely withinthe past year	Performed this skill safely oncetime within the past year	Performed this skill safely on morethan one occasion within the past	Not at all		somewhat		Very confident
							1	2	3	4	5
equipment for responding											
ii. Recognize situations requiring expertise beyondmidwifery care											
iii. Maintain communication with women about nature of problem, actions taken, and											
iv. Determine the need for immediate intervention and respond											
v. Implement timely and appropriate intervention, inter-professional consultation and/or timelyreferral taking											
vi. Provide accurate oral andwritten information to other care providers whenreferral is made.											
vii. Collaborate with decision-making if possible and											
**COMPETENCY 1.13 Care for women who experience physical and sexual violence and**											
**a) KNOWLEDGE**											
i. Socio-cultural, behavioural, and											

ICM Essential Competencies	COMPETENCE						CONFIDENCE				
	Knowledge (e.g. CPDs, guidelines)			Skill and Behaviour			Self-rating of confidence in current knowledge OR				
Knowledge, Skill, and Behaviour	Not updated my knowledge(>3years)	Updated my knowledge (>2-3 years)	Current in my knowledge (within 1year)	Not performed this skill safely withinthe past year	Performed this skill safely oncetime within the past year	Performed this skill safely on morethan one occasion within the past	Not at all		somewhat		Very confident
							1	2	3	4	5
conditions that often accompany violence andabuse											
ii. Resources in communityto assist women and children											
iii. Risks of disclosure											
iv. Effects of gender-based violence, emotional abuse, and physical neglect											
**a) SKILLS & BEHAVIOURS**											
i. Protect privacy andconfidentiality											
ii. Provide information to allwomen about sources of help regardless of whether there is disclosure											
iii. Inquire routinely aboutsafety at home,											
iv. Recognize potential signsof abuse from physical appearance, emotional affect, related risk behaviours such as substance											
v. Provide special support foradolescents and											

ICM Essential Competencies	COMPETENCE						CONFIDENCE				
	Knowledge (e.g. CPDs, guidelines)			Skill and Behaviour							
Knowledge, Skill, and Behaviour	Not updated my knowledge(>3years)	Updated my knowledge (>2-3 years)	Current in my knowledge (within 1year)	Not performed this skill safely withinthe past year	Performed this skill safely oncetime within the past year	Performed this skill safely on morethan one occasion within the past	Self-rating of confidence in current knowledge OR				
							Not at all		somewhat		Very confident
							1	2	3	4	5
gender-based violence including rape											
vi. Refer to community resources, assist in locating safe setting asneeded											
**CATEGORY 2: PRE-PREGNANCY AND ANTENATAL**											
**COMPETENCY 2.1 Provide pre-pregnancy care**											
**a) KNOWLEDGE**											
i. Anatomy and physiology of female and male relatedto reproduction and											
ii. Socio-cultural aspects ofhuman sexuality											
iii. Evidence based screeningfor cancer of reproductiveorgans and other health											
**b) SKILLS & BEHAVIOURS**											
i. Identify and assist in reducing barriers related toaccessing care and using sexual and reproductive health											
ii. Assess nutritional status, current immunization status, health behaviours											

ICM Essential Competencies	COMPETENCE						CONFIDENCE				
	Knowledge (e.g. CPDs, guidelines)			Skill and Behaviour			Self-rating of confidence in current knowledge OR				
Knowledge, Skill, and Behaviour	Not updated my knowledge(>3years)	Updated my knowledge (>2-3 years)	Current in my knowledge (within 1year)	Not performed this skill safely withinthe past year	Performed this skill safely oncetime within the past year	Performed this skill safely on morethan one occasion within the past	Not at all		somewhat		Very confident
							1	2	3	4	5
medical conditions, and exposure to known											
iii. Carry out screening procedures for sexually transmitted and other infections, HIV, cervicalcancer											
iv. Provide counselling about nutritional supplements such as folic acid, dietary intake, exercise, updating immunizations as needed, modifying risk behaviours,											
**COMPETENCY 2.2 Assess foetal well-being**											
**a) KNOWLEDGE**											
i. Placental physiology, embryology, foetal growth and development, and indicators of foetal well-being											
ii. Evidence-based guidelines for use ofultrasound											

ICM Essential Competencies	COMPETENCE						CONFIDENCE				
	Knowledge (e.g. CPDs, guidelines)			Skill and Behaviour							
Knowledge, Skill, and Behaviour	Not updated my knowledge(>3years)	Updated my knowledge (>2-3 years)	Current in my knowledge (within 1year)	Not performed this skill safely withinthe past year	Performed this skill safely oncetime within the past year	Performed this skill safely on morethan one occasion within the past	Self-rating of confidence in current knowledge OR				
							Not at all		somewhat		Very confident
							1	2	3	4	5
**b) SKILLS & BEHAVIOURS**											
i. Assess foetal size, amniotic fluid volume, foetal position, activity, and heart rate from examination of maternalabdomen											
ii. Determine whether thereare indications for additional assessment/examination											
iii. Assess foetal movementsand ask woman about foetal											
**COMPETENCY 2.3 Monitor the progression of pregnancy**											
**a) KNOWLEDGE**											
i. Usual physiological andphysical changes with advancing											
ii. Nutritional requirementsof											
iii. Common psychological responses to pregnancyand											
iv. Evidence informed antenatal care policies andguidelines,											

ICM Essential Competencies	COMPETENCE						CONFIDENCE				
	Knowledge (e.g. CPDs, guidelines)			Skill and Behaviour			Self-rating of confidence in current knowledge OR				
Knowledge, Skill, and Behaviour	Not updated my knowledge(>3years)	Updated my knowledge (>2-3 years)	Current in my knowledge (within 1year)	Not performed this skill safely withinthe past year	Performed this skill safely oncetime within the past year	Performed this skill safely on morethan one occasion within the past	Not at all		somewhat		Very confident
							1	2	3	4	5
frequency of antenatal visits											
**b) SKILLS & BEHAVIOURS**											
i. Conduct assessments throughout pregnancy of woman's physical and psychological well-being,family relationships, and											
ii. Provide information regarding normal pregnancy to woman, herpartner, family members,or other											
iii. Suggest measures to cope with common discomforts of											
iv. Provide information (including written and/ or pictorial) about danger signs, (e.g., vaginal bleeding, signs of pretermlabour, pre labour, ruptureof membranes) emergencyprepared-											
v. Review findings and revise plan of care withwoman as pregnancy											

ICM Essential Competencies	COMPETENCE						CONFIDENCE				
	Knowledge (e.g. CPDs, guidelines)			Skill and Behaviour							
Knowledge, Skill, and Behaviour	Not updated my knowledge(>3years)	Updated my knowledge (>2-3 years)	Current in my knowledge (within 1year)	Not performed this skill safely withinthe past year	Performed this skill safely oncetime within the past year	Performed this skill safely on morethan one occasion within the past	Self-rating of confidence in current knowledge OR				
							Not at all		somewhat		Very confident
							1	2	3	4	5
**COMPETENCY 2.4 Promote and support health behaviours that improve well being**											
**a) KNOWLEDGE**											
i. Impact of adverse social,environmental, and economic conditions on											
ii. Effects of inadequate nutrition and heavy											
iii. Effects of tobacco use andexposure to second-hand smoke, use of alcohol and											
iv. Effects of prescribed											
v. Community resources forincome support, food access, and programs to minimize risks of substance											
vi. Strategies to prevent or reduce risks of mother-to-child disease transmission including infant feeding											
vii. Effects of gender-based violence, emotional abuse,and physical neglect											

ICM Essential Competencies	COMPETENCE						CONFIDENCE				
	Knowledge (e.g. CPDs, guidelines)			Skill and Behaviour			Self-rating of confidence in current knowledge OR				
Knowledge, Skill, and Behaviour	Not updated my knowledge(>3years)	Updated my knowledge (>2-3 years)	Current in my knowledge (within 1year)	Not performed this skill safely withinthe past year	Performed this skill safely oncetime within the past year	Performed this skill safely on morethan one occasion within the past	Not at all		somewhat		Very confident
							1	2	3	4	5
**b) SKILLS & BEHAVIOURS**											
i. Provide emotional supportto women to encourage change in											
ii. Provide information to woman and family aboutimpact on mother and foetus of											
iii. Counsel women about andoffer referral to appropriate persons or agencies for assistance andtreatment											
iv. Respect women's decisions about participating in treatmentsand											
v. Make recommendations and identify re- sources forsubstance abuse reduction/cessation in pregnancy											
**COMPETENCY 2.5 Provide anticipatory guidance related to pregnancy, birth, breastfeeding parenthood, and change in the family**											
**a) KNOWLEDGE**											
i. Needs of Individuals andfamilies for different information at											

ICM Essential Competencies	COMPETENCE						CONFIDENCE				
	Knowledge (e.g. CPDs, guidelines)			Skill and Behaviour			Self-rating of confidence in current knowledge OR				
Knowledge, Skill, and Behaviour	Not updated my knowledge(>3years)	Updated my knowledge (>2-3 years)	Current in my knowledge (within 1year)	Not performed this skill safely withinthe past year	Performed this skill safely oncetime within the past year	Performed this skill safely on morethan one occasion within the past	Not at all		somewhat		Very confident
							1	2	3	4	5
times in their respective life cycles											
ii. Methods of providing information to individualsand groups											
iii. Methods of eliciting maternal feelings andexpectations for self, infant, and											
**b) SKILLS & BEHAVIOURS**											
i. Participate in--and referwomen and support persons to--childbirth education											
ii. Convey information accurately and clearly andrespond to needs of individuals											
iii. Prepare the woman, partner, and family to recognize labour onset,when to seek care, and											
iv. Provide information aboutpostpartum needs including contraception, care of newborn infants, and the importance of exclusive breast											

ICM Essential Competencies	COMPETENCE						CONFIDENCE				
	Knowledge (e.g. CPDs, guidelines)			Skill and Behaviour							
Knowledge, Skill, and Behaviour	Not updated my knowledge(>3years)	Updated my knowledge (>2-3 years)	Current in my knowledge (within 1year)	Not performed this skill safely withinthe past year	Performed this skill safely oncetime within the past year	Performed this skill safely on morethan one occasion within the past	Self-rating of confidence in current knowledge OR				
							Not at all		somewhat		Very confident
							1	2	3	4	5
v. Identify needs or problemsrequiring further expertise or referral such as excessive fear, and dysfunctional											
**COMPETENCY 2.6 Detect, manage, and refer women with complicated pregnancies**											
**a) KNOWLEDGE**											
i. Complications of early pregnancy such as threatened or actual miscarriage, and											
ii. Foetal compromise, growth restriction, malposition,											
iii. Signs and symptoms ofmaternal pathologic conditions such as pre-eclampsia, gestational diabetes,											
iv. Signs of acute emergencies such as haemorrhage, seizures,and sepsis											
**b) SKILLS & BEHAVIOURS**											

ICM Essential Competencies	COMPETENCE						CONFIDENCE				
	Knowledge (e.g. CPDs, guidelines)			Skill and Behaviour			Self-rating of confidence in current knowledge OR				
Knowledge, Skill, and Behaviour	Not updated my knowledge(>3years)	Updated my knowledge (>2-3 years)	Current in my knowledge (within 1year)	Not performed this skill safely withinthe past year	Performed this skill safely oncetime within the past year	Performed this skill safely on morethan one occasion within the past	Not at all		somewhat		Very confident
							1	2	3	4	5
i. Collaborate in care of orrefer for treatment of complications											
ii. Implement critical care activities to support vitalbody functions (e.g.											
iii. Mobilize blood donors ifnecessary											
iv. Transfer to higher levelfacility if needed											
**COMPETENCY 2.7 Assist the woman and her family to plan for an appropriate place of**											
**a) KNOWLEDGE**											
i. Evidence about birth outcomes in different birthplace											
ii. Availability of options inspecific location; limitations of climate, geography, means of transport, and resources											
iii. Local policies andguidelines											
**b) SKILLS & BEHAVIOURS**											
i. Discuss options, preferences and contingency plans withwoman and											

ICM Essential Competencies	COMPETENCE						CONFIDENCE				
	Knowledge (e.g. CPDs, guidelines)			Skill and Behaviour							
Knowledge, Skill, and Behaviour	Not updated my knowledge(>3years)	Updated my knowledge (>2-3 years)	Current in my knowledge (within 1year)	Not performed this skill safely withinthe past year	Performed this skill safely oncetime within the past year	Performed this skill safely on morethan one occasion within the past	Self-rating of confidence in current knowledge OR				
							Not at all		somewhat		Very confident
							1	2	3	4	5
persons and respect their											
ii. Provide information aboutpreparing birth site if in community, e.g., travel and											
iii. Promote the availability ofa full range of birth settings											
**COMPETENCY 2.8 Provide care to women with unintended or mistimed pregnancy**											
**a) KNOWLEDGE**											
i. Complexity of decision- making about unintendedor											
ii. Emergency											
iii. Legal options for induced abortion; eligibility and availability of medical andsurgical											
iv. Medications used to induce abortion; properties, effects, and											
v. Risks of unsafe abortion											
vi. Family planning methodsappropriate for the post- abortion											

ICM Essential Competencies	COMPETENCE						CONFIDENCE				
	Knowledge (e.g. CPDs, guidelines)			Skill and Behaviour			Self-rating of confidence in current knowledge OR				
Knowledge, Skill, and Behaviour	Not updated my knowledge(>3years)	Updated my knowledge (>2-3 years)	Current in my knowledge (within 1year)	Not performed this skill safely withinthe past year	Performed this skill safely oncetime within the past year	Performed this skill safely on morethan one occasion within the past	Not at all		somewhat		Very confident
							1	2	3	4	5
vii. Care and support (physicaland psychological) for Post											
**b) SKILLS & BEHAVIOURS**											
i. Confirm pregnancy and determine gestational age;refer for ultrasound if unknown gestation and/orsymptoms of ectopic											
ii. Counsel woman about options to maintain or endthe pregnancy and respectthe ultimate											
iii. Provide supportive antenatal care if pregnancycontinued; refer to agencies, and social services for support and assistance											
iv. Identify from obstetric, medical, and social history, contraindications to medication or											
v. Provide information aboutlegal regulations, eligibility, and access to abortion services											

ICM Essential Competencies	COMPETENCE						CONFIDENCE				
	Knowledge (e.g. CPDs, guidelines)			Skill and Behaviour							
Knowledge, Skill, and Behaviour	Not updated my knowledge(>3years)	Updated my knowledge (>2-3 years)	Current in my knowledge (within 1year)	Not performed this skill safely withinthe past year	Performed this skill safely oncetime within the past year	Performed this skill safely on morethan one occasion within the past	Self-rating of confidence in current knowledge OR				
							Not at all		somewhat		Very confident
							1	2	3	4	5
vi. Provide information about abortion procedures, potential complications, management of pain, and when to seek help											
vii. Refer to provider of abortion services uponrequest											
viii. Provide post-abortion											
ix. Confirm expulsion of products of conception from history, ultrasound,or											
x. Review options for contraception and initiateimmediate use											
xi. Explore psychological response to abortion											
**CATEGORY 3: CARE DURING LABOUR AND BIRTH**											
**COMPETENCY 3.1 Promote physiologic labour and birth**											
**a) KNOWLEDGE**											
i. Anatomy of maternal pelvis and foetus; mechanisms of labour fordifferent foetal presentations											
ii. Physiologic onset andprogression of											

ICM Essential Competencies	COMPETENCE						CONFIDENCE				
	Knowledge (e.g. CPDs, guidelines)			Skill and Behaviour							
Knowledge, Skill, and Behaviour	Not updated my knowledge(>3years)	Updated my knowledge (>2-3 years)	Current in my knowledge (within 1year)	Not performed this skill safely withinthe past year	Performed this skill safely oncetime within the past year	Performed this skill safely on morethan one occasion within the past	Self-rating of confidence in current knowledge OR				
							Not at all		somewhat		Very confident
							1	2	3	4	5
iii. Evidence about interventions in normallabour and											
iv. Cultural and social beliefsand traditions											
v. Signs and behaviours oflabour progress; factorsthat impede labour progress											
vi. Methods of assessingfoetus											
**b) SKILLS & BEHAVIOURS**											
i. Provide care for a womanin the birth setting of her choice, following policiesand											
ii. Obtain relevant obstetric, medical, and											
iii. Perform and interpret focused physical examination of the woman											
iv. Order and interpret laboratory tests if											
v. Assess woman's physical and behavioural responses											
vi. Provide information, support, and encouragement to											

ICM Essential Competencies	COMPETENCE						CONFIDENCE				
	Knowledge (e.g. CPDs, guidelines)			Skill and Behaviour			Self-rating of confidence in current knowledge OR				
Knowledge, Skill, and Behaviour	Not updated my knowledge(>3years)	Updated my knowledge (>2-3 years)	Current in my knowledge (within 1year)	Not performed this skill safely withinthe past year	Performed this skill safely oncetime within the past year	Performed this skill safely on morethan one occasion within the past	Not at all		somewhat		Very confident
							1	2	3	4	5
and support persons throughout											
vii. Provide respectful one-to-one care											
viii. Encourage freedom of movement and uprightpositions											
ix. Provide nourishment and fluids											
x. Offer and support woman to use strategies for coping with labour pain, e.g., controlled breathing, waterimmersion, relaxation, massage, and											
xi. Assess regularly parameters of maternal- foetal status, and e.g. vitalsigns, contractions, cervical changes, and											
xii. Use labour progress graphic display to recordfindings and assist in detecting labour delay orother											
xiii. Augment uterine contractility											

ICM Essential Competencies	COMPETENCE						CONFIDENCE				
	Knowledge (e.g. CPDs, guidelines)			Skill and Behaviour			Self-rating of confidence in current knowledge OR				
Knowledge, Skill, and Behaviour	Not updated my knowledge(>3years)	Updated my knowledge (>2-3 years)	Current in my knowledge (within 1year)	Not performed this skill safely withinthe past year	Performed this skill safely oncetime within the past year	Performed this skill safely on morethan one occasion within the past	Not at all		somewhat		Very confident
							1	2	3	4	5
using non-pharmacological or pharmacological agents to prevent non-											
xiv. Prevent unnecessary routine interventions, e.g.,amniotomy, electronic foetal monitoring, directed											
**COMPETENCY 3.2 Manage a safe spontaneous vaginal birth and prevent complications**											
**a) KNOWLEDGE**											
i. Methods of conducting birth to protect perineal											
ii. Evidence about conduct ofthird stage, including use of uterotonics											
iii. Potential complicationsand their immediate treatment e.g., shoulder											
iv. Signs of placental separation; appearance of normal placenta, membranes, and umbilicalcord											

ICM Essential Competencies	COMPETENCE						CONFIDENCE				
	Knowledge (e.g. CPDs, guidelines)			Skill and Behaviour			Self-rating of confidence in current knowledge OR				
Knowledge, Skill, and Behaviour	Not updated my knowledge(>3years)	Updated my knowledge (>2-3 years)	Current in my knowledge (within 1year)	Not performed this skill safely withinthe past year	Performed this skill safely oncetime within the past year	Performed this skill safely on morethan one occasion within the past	Not at all		somewhat		Very confident
							1	2	3	4	5
v. Types of perineal and vaginal trauma requiringrepair and suturing techniques											
**b) SKILLS & BEHAVIOURS**											
i. Support the woman to givebirth in her position of choice											
ii. Ensure presence of clean necessary supplies andsource of											
iii. Coach woman about pushing to control expulsion of presentingpart, avoid routine episiotomy											
iv. Undertake appropriate manoeuvres and use maternal position to facilitate vertex, face, orbreech birth											
v. Delay cord clamping											
vi. Manage nuchal cord											
vii. Assess immediate condition of											
viii. Provide skin to skin contact and warm environment											

ICM Essential Competencies	COMPETENCE						CONFIDENCE				
	Knowledge (e.g. CPDs, guidelines)			Skill and Behaviour			Self-rating of confidence in current knowledge OR				
Knowledge, Skill, and Behaviour	Not updated my knowledge(>3years)	Updated my knowledge (>2-3 years)	Current in my knowledge (within 1year)	Not performed this skill safely withinthe past year	Performed this skill safely oncetime within the past year	Performed this skill safely on morethan one occasion within the past	Not at all		somewhat		Very confident
							1	2	3	4	5
ix. Deliver placenta and membranes and inspect forcompleteness											
x. Assess uterine tone, maintain firm contraction,and estimate and record											
xi. Inspect vaginal and perineal areas for trauma,and repair as needed, following policies											
**CATEGORY 4: ONGOING CARE OF WOMEN AND NEWBORNS**											
**COMPETENCY 4.1 Provide care to healthy newborn infant**											
**a) KNOWLEDGE**											
i. Appearance and behaviourof infant in early life; cardio-respiratory changes related to adapting to											
ii. Growth and development in initial weeks andmonths of life											
iii. Protocols for screening formetabolic conditions, infectious conditions, and congenital											
iv. Protocols/guidelines for immunizations in											

ICM Essential Competencies	COMPETENCE						CONFIDENCE				
	Knowledge (e.g. CPDs, guidelines)			Skill and Behaviour			Self-rating of confidence in current knowledge OR				
Knowledge, Skill, and Behaviour	Not updated my knowledge(>3years)	Updated my knowledge (>2-3 years)	Current in my knowledge (within 1year)	Not performed this skill safely withinthe past year	Performed this skill safely oncetime within the past year	Performed this skill safely on morethan one occasion within the past	Not at all		somewhat		Very confident
							1	2	3	4	5
v. Evidence-based information about infantcircumcision; family values, beliefs, and cultural norms											
**b) SKILLS & BEHAVIOURS**											
i. Examine infant at frequentintervals to monitor growth and developmental											
ii. Distinguish normal variation in newborn appearance and behaviourfrom those indicating pathologic											
iii. Administer immunizations, carry out screening tests as											
iv. Provide information to parents about a safe environment for infant, frequent feeding, care of umbilical cord, voiding and stooling,											
**COMPETENCY 4.2 Provide postnatal care for the healthy woman**											
**a) KNOWLEDGE**											

ICM Essential Competencies	COMPETENCE						CONFIDENCE				
	Knowledge (e.g. CPDs, guidelines)			Skill and Behaviour			Self-rating of confidence in current knowledge OR				
Knowledge, Skill, and Behaviour	Not updated my knowledge(>3years)	Updated my knowledge (>2-3 years)	Current in my knowledge (within 1year)	Not performed this skill safely withinthe past year	Performed this skill safely oncetime within the past year	Performed this skill safely on morethan one occasion within the past	Not at all		somewhat		Very confident
							1	2	3	4	5
i. Physiological changes following birth, uterine involution, onset of lactation, healing of											
ii. Common discomforts ofthe postnatal period andcomfort measures											
iii. Need for rest, support, andnutrition to support lactation											
iv. Psychological responses to mothering role, addition of infant to											
**b) SKILLS & BEHAVIOURS**											
i. Review history of pregnancy, labour, andbirth											
ii. Conduct a focused physical exam to assess breast changes and involution. Monitor blood loss and other											
iii. Assess mood and feelingsabout motherhood and											
iv. Provide pain control strategies if needed											

ICM Essential Competencies	COMPETENCE						CONFIDENCE				
	Knowledge (e.g. CPDs, guidelines)			Skill and Behaviour			Self-rating of confidence in current knowledge OR				
Knowledge, Skill, and Behaviour	Not updated my knowledge(>3years)	Updated my knowledge (>2-3 years)	Current in my knowledge (within 1year)	Not performed this skill safely withinthe past year	Performed this skill safely oncetime within the past year	Performed this skill safely on morethan one occasion within the past	Not at all		somewhat		Very confident
							1	2	3	4	5
uterine contractions, and perineal trauma											
v. Provide information aboutself-care that enables mother to meet needs of newborn, e.g. adequate food, nutritional supplements, usual activities, rest periods, andhousehold											
vi. Provide information aboutsafe sex, family planning methods appropriate for the immediate postnatal period, and pregnancy											
vii. Provide pain control strategies if needed for uterine contractions, and											
**COMPETENCY 4.3 Detect and treat or refer postnatal complications in woman and newborn infant**											
**a) KNOWLEDGE**											
i. Signs and symptoms of conditions in the postnatalperiod that may respond toearly intervention (e.g. sub-involution, anaemia, and urinary retention)											

ICM Essential Competencies	COMPETENCE						CONFIDENCE				
	Knowledge (e.g. CPDs, guidelines)			Skill and Behaviour			Self-rating of confidence in current knowledge OR				
Knowledge, Skill, and Behaviour	Not updated my knowledge(>3years)	Updated my knowledge (>2-3 years)	Current in my knowledge (within 1year)	Not performed this skill safely withinthe past year	Performed this skill safely oncetime within the past year	Performed this skill safely on morethan one occasion within the past	Not at all		somewhat		Very confident
							1	2	3	4	5
referral to more specialized provider or facility (e.g. hematoma, thrombophlebitis, sepsis, obstetric fistula, and incontinence) life threatening complicationsrequiring immediate response and specialized care (haemorrhage, amniotic fluid											
ii. Signs and symptoms of postnatal depression, anxiety, and psychosis											
iii. Bereavement care following perinatal											
iv. Congenital anomalies, andgenetic conditions											
v. Needs of pre-term and lowbirth weight infants											
vi. Symptoms and treatment of withdrawal frommaternal											
vii. Prevention of mother-to-child transmission of infections such as HIV, hepatitis B and C											

ICM Essential Competencies	COMPETENCE						CONFIDENCE				
	Knowledge (e.g. CPDs, guidelines)			Skill and Behaviour							
Knowledge, Skill, and Behaviour	Not updated my knowledge(>3years)	Updated my knowledge (>2-3 years)	Current in my knowledge (within 1year)	Not performed this skill safely withinthe past year	Performed this skill safely oncetime within the past year	Performed this skill safely on morethan one occasion within the past	Self-rating of confidence in current knowledge OR				
							Not at all		somewhat		Very confident
							1	2	3	4	5
viii. Signs and symptoms of common health problemsand their											
**b) SKILLS & BEHAVIOURS**											
i. Provide information to woman and family aboutpotential complications and											
ii. Assess woman during postnatal period to detect signs and symptoms											
iii. Distinguish postnatal depression from transient anxiety about caring for baby, assess availability ofhelp and support at home, and provide emotional											
iv. Provide counselling and follow-up care for womenand family members who experience stillbirth, neonatal death, serious infant illness, and											
v. Provide first line measuresto treat or stabilize identified											

ICM Essential Competencies	COMPETENCE						CONFIDENCE				
	Knowledge (e.g. CPDs, guidelines)			Skill and Behaviour			Self-rating of confidence in current knowledge OR				
Knowledge, Skill, and Behaviour	Not updated my knowledge(>3years)	Updated my knowledge (>2-3 years)	Current in my knowledge (within 1year)	Not performed this skill safely withinthe past year	Performed this skill safely oncetime within the past year	Performed this skill safely on morethan one occasion within the past	Not at all		somewhat		Very confident
							1	2	3	4	5
vi. Arrange referral and/ortransfer as											
**COMPETENCY 4.4 Promote and support breastfeeding**											
**a) KNOWLEDGE**											
i. Physiology of lactation											
ii. Nutritional needs of newborn infants, includinglow birth											
iii. Social, psychological, andcultural aspects of breastfeeding											
iv. Evidence about benefits ofbreastfeeding											
v. Indications and contraindications to use ofdrugs and substances during											
vi. Awareness of lactation aids											
**b) SKILLS & BEHAVIOURS**											
i. Promote early and exclusive breastfeeding while respecting a woman' s choice regardingnewborn											
ii. Provide information about infant needs, frequency											

ICM Essential Competencies	COMPETENCE						CONFIDENCE				
	Knowledge (e.g. CPDs, guidelines)			Skill and Behaviour			Self-rating of confidence in current knowledge OR				
Knowledge, Skill, and Behaviour	Not updated my knowledge(>3years)	Updated my knowledge (>2-3 years)	Current in my knowledge (within 1year)	Not performed this skill safely withinthe past year	Performed this skill safely oncetime within the past year	Performed this skill safely on morethan one occasion within the past	Not at all		somewhat		Very confident
							1	2	3	4	5
and duration of feedings, and weight gain											
iii. Identify and manage breastfeeding problems(e.g., mastitis, low milk supply,											
iv. Provide information to women breastfeeding multiple newborns											
v. Refer women to breastfeeding support asindicated											
vi. Advocate for breastfeeding in familyand											
vii. Identify and manage breastfeeding problems(e.g., mastitis, low milk supply,											
**COMPETENCY 4.5 Provide family planning services**											
**a) KNOWLEDGE**											
i. Anatomy and physiology of female and male relatedto reproduction and sexualdevelopment											
ii. Socio-cultural aspects of human sexuality											

ICM Essential Competencies	COMPETENCE						CONFIDENCE				
	Knowledge (e.g. CPDs, guidelines)			Skill and Behaviour							
Knowledge, Skill, and Behaviour	Not updated my knowledge(>3years)	Updated my knowledge (>2-3 years)	Current in my knowledge (within 1year)	Not performed this skill safely withinthe past year	Performed this skill safely oncetime within the past year	Performed this skill safely on morethan one occasion within the past	Self-rating of confidence in current knowledge OR				
							Not at all		somewhat		Very confident
							1	2	3	4	5
iii. Family planning methods including natural, barrier, hormonal, implantable; emergency contraception, sterilization; their possibleside effects,											
iv. Available written and pictorial resources for teaching about family											
v. Pregnancy options for HIVpositive women or											
**b) SKILLS & BEHAVIOURS**											
i. Provide and protect privacy and confidentialityfor discussions about family planning knowledge, goals for limiting and/or spacing of children, and											
ii. Obtain relevant history ofuse of methods, medical conditions, socio- culturalvalues, and preferences that influence choice of											

ICM Essential Competencies	COMPETENCE						CONFIDENCE				
	Knowledge (e.g. CPDs, guidelines)			Skill and Behaviour			Self-rating of confidence in current knowledge OR				
Knowledge, Skill, and Behaviour	Not updated my knowledge(>3years)	Updated my knowledge (>2-3 years)	Current in my knowledge (within 1year)	Not performed this skill safely withinthe past year	Performed this skill safely oncetime within the past year	Performed this skill safely on morethan one occasion within the past	Not at all		somewhat		Very confident
							1	2	3	4	5
iii. Provide information abouthow to use, effectiveness, and cost of various methods to support informed											
iv. Provide methods according to scope of practice and protocols, orrefer to another											
v. Provide follow-up assessment of use, satisfaction, and											
vi. Refer for woman or partner for sterilization											
